# Is the Volume of the Caudate Nuclei Associated With Area of Secondary Hyperalgesia? – Protocol for a 3-Tesla MRI Study of Healthy Volunteers

**DOI:** 10.2196/resprot.5680

**Published:** 2016-06-17

**Authors:** Morten Sejer Hansen, Mohammad Sohail Asghar, Jørn Wetterslev, Christian Bressen Pipper, Johan Johan Mårtensson, Lino Becerra, Anders Christensen, Janus Damm Nybing, Inger Havsteen, Mikael Boesen, Jørgen Berg Dahl

**Affiliations:** ^1^Department of Anaesthesiology, 4231Centre of head and orthopaedicsRigshospitalet, Copenhagen University HospitalCopenhagenDenmark; ^2^Copenhagen Trial Unit, Centre for Clinical Intervention ResearchDepartment 7812CopenhagenDenmark; ^3^Copenhagen UniversityFaculty of health, Section of biostatisticsCopenhagenDenmark; ^4^Lund UniversityDepartment of psychologyLundSweden; ^5^Childrens HospitalHarvard UniversityBoston, MAUnited States; ^6^Department of RadiologyCopenhagen University Hospital, Bispebjerg and Frederiksberg HospitalsCopenhagenDenmark; ^7^Department of Anaesthesiology, dep. ZCopenhagen University Hospital, Bispebjerg and Frederiksberg HospitalsCopenhagenDenmark

**Keywords:** Pain, Magnetic resonance imaging, Hyperalgesia, Central sensitization, Quantitative sensory testing, Anaesthesiology, Physiology

## Abstract

**Background:**

Experience and development of pain may be influenced by a number of physiological, psychological, and psychosocial factors. In a previous study we found differences in neuronal activation to noxious stimulation, and microstructural neuroanatomical differences, when comparing healthy volunteers with differences in size of the area of secondary hyperalgesia following a standardized burn injury.

**Objective:**

We aim to investigate the degree of association between the volume of pain-relevant structures in the brain and the size of the area of secondary hyperalgesia following brief thermal sensitization.

**Methods:**

The study consists of one experimental day, in which whole-brain magnetic resonance imaging (MRI) scans will be conducted including T1-weighed three-dimensional anatomy scan, diffusion tensor imaging, and resting state functional MRI. Before the experimental day, all included participants will undergo experimental pain testing in a parallel study (Clinicaltrials.gov Identifier: NCT02527395). Results from this experimental pain testing, as well as the size of the area of secondary hyperalgesia from the included participants, will be extracted from this parallel study.

**Results:**

The association between the volume of pain-relevant structures in the brain and the area of secondary hyperalgesia will be investigated by linear regression of the estimated best linear unbiased predictors on the individual volumes of the pain relevant brain structures.

**Conclusions:**

We plan to investigate the association between experimental pain testing parameters and the volume, connectivity, and resting state activity of pain-relevant structures in the brain. These results may improve our knowledge of the mechanisms responsible for the development of acute and chronic pain.

**ClinicalTrial:**

Danish Research Ethics Committee (identifier: H-15010473). Danish Data Protection Agency (identifier: RH-2015-149). Clinicaltrials.gov NCT02567318; http://clinicaltrials.gov/ct2/show/NCT02567318 (Archived by WebCite at http://www.webcitation.org/6i4OtP0Oi)

## Introduction

The experience and development of pain may be influenced by a number of physiological, psychological, and psychosocial factors. However, our knowledge of the mechanisms responsible for acute and chronic pain is still not complete. A standardized burn injury in the skin provokes reversible primary and secondary hyperalgesia in healthy volunteers. Injury-induced primary hyperalgesia is located in the traumatized area and is characterized by reduced thresholds for thermal and mechanical stimulation. Secondary hyperalgesia is located around the traumatized area, and is characterized by reduced thresholds for mechanical stimulation [1,2].

Previous studies have demonstrated that secondary hyperalgesia to punctate mechanical stimuli is a result of changes in the central nervous system, in part due to sensitization in response to nerve signals by A-fiber nociceptors [1,3-5]. Secondary hyperalgesia can be provoked by a number of different conditioning stimuli, and according to previous studies this is a robust phenomenon that can be applied to investigate basic pain physiology [2,6,7]. Central neuronal sensitization is presumed to play an important role in a number of different pain conditions, such as osteoarthritis, fibromyalgia, and postoperative pain [3].

To investigate the basic physiological mechanisms of pain, our research group has worked extensively with experimental physiological pain models [8-10]. In previous studies, we found indications of large inter-individual variations in the size of the area of secondary hyperalgesia following identical experimental pain stimulation. Moreover, we observed that the area of secondary hyperalgesia may be an identifiable phenotypic indicator and predictor of individual pain responses [10,11].

To further explore the pathophysiology of central sensitization, we first investigated functional changes in the brain by magnetic resonance imaging (MRI) after induction of secondary hyperalgesia by a first degree burn injury. The first degree burn injury was induced 100 minutes before initiation of MRI, and interestingly we found differences in neuronal activation to noxious stimulation in healthy volunteers that developed either large or small areas of secondary hyperalgesia. These findings suggest differences in central pain processing related to phenotypical expression of secondary hyperalgesia. Furthermore, we found indications of differences in the volumes of the caudate nuclei, as well as other microstructural neuroanatomical differences between the two groups [11], encouraging further studies with larger sample sizes to elucidate the importance of each finding in the pathophysiology of pain and central sensitization.

The basal ganglia have proved to be essential in the pain processing in humans [12-14]. Moreover, the role of the caudate nuclei in the processing and modulation of pain have been investigated in human [12-27] as well as animal [28-39] studies. The caudate nuclei are believed to play a role in integration and control of sensory, motor, and motivational information, and thus essential in coordinating behavioral responses [14]. The caudate nuclei have also been demonstrated to be involved in the sensory processing and spatial location of noxious stimuli [24], in the suppression and modulation of pain [26], and are activated during pain expectancy [40].

Reduced grey matter volume of the caudate nuclei has been demonstrated in patients with trigeminal neuralgia [20], knee osteoarthritis [22], lumbar disc herniation [21], and migraines [15,27]. Moreover, reduced regional cerebral blood flow in the caudate nuclei has been seen in patients with fibromyalgia [23], familial restless legs syndrome [25], and chronic fatigue syndrome [16]. Thus, the caudate nuclei are essential sites of pain processing, and may play a role in the development of secondary hyperalgesia, and consequently in the processes involved with central sensitization.

A recent study confirmed that areas of secondary hyperalgesia have a low intra-individual variation compared to the inter-individual variation [41]. This finding enables us to phenotype healthy volunteers based on the size of the area of secondary hyperalgesia. It remains uncertain whether these phenotypes are related to other pain syndromes or entities, are involved in the risk of developing chronic pain, or influence factors in the individual’s pain sensitivity. Likewise, it is unclear if important structures such as the caudate nuclei (or other CNS differences) are closely related to secondary hyperalgesia phenotyping, warranting more research in this area.

With this study we aim to investigate the association between the volume of pre-defined pain-relevant structures in the brain and the size of the areas of secondary hyperalgesia.

## Methods

### Study Design

This study is a confirmatory study designed to investigate the association between the size of the area of secondary hyperalgesia following brief thermal sensitization (BTS; see *Clinical Evaluations*) and the volume of the caudate nuclei in healthy volunteers. On the experimental day, a whole brain MRI scan will be conducted including T1-weighed three-dimensional (3D) anatomy scan, diffusion tensor imaging, and resting-state functional MRI (rs-fMRI), in consecutive sequence (see *Clinical Evaluations*). No other tests or assessments will be performed on the experimental day. The total duration of the experimental day is approximately 50 minutes.

Before the experimental day, all included participants will undergo pain testing in a parallel study (Clinicaltrials.gov Identifier: NCT02527395). Results from the pain testing, as well as the size of the area of secondary hyperalgesia from the included participants, will be extracted from this parallel study. The experimental day will be conducted at a maximum of 2 months and a minimum of 2 weeks after the completion of the BTS and additional pain testing.

### Setting

The MRI scans will be conducted at the Department of Radiology, Bispebjerg and Frederiksberg Hospitals, Copenhagen, Denmark. The data analysis will be conducted at the Department of Anesthesiology, Centre of Head and Orthopedics, Rigshospitalet, Copenhagen, Denmark.

### Study Participants

Healthy male volunteers will be included in the study. The study participants will receive information regarding possible risks and side effects, and will be provided with written and oral information concerning the study. The participants will receive €67 (US $74) for their participation in the study. Inclusion and exclusion criteria are presented in Table 1.

**Table 1:** Inclusion, and exclusion criteria.

Inclusion criteria	Exclusion criteria
Age ≥18 years and ≤35 years	Study participants who cannot cooperate to the test
Male sex	Study participants with a substance abuse, assessed by the investigator
Study participants who have a weekly intake of >21 units of alcohol, or a have consumed >3 units of alcohol within 24 hours before experimental day Speak and understand the Danish language Signed informed consent Have participated and completed the study: “*To determine the degree of association between Heat Pain Detection Threshold and area of secondary hyperalgesia following Brief Thermal Sensitization in healthy male volunteers”* (Clinicaltrials.gov Identifier: NCT02527395)	Study participants who have consumed analgesics within 3 days before experimental day Study participants who have consumed antihistamines within 48 hours before experimental day Study participants who have consumed antidepressant medication within 30 days before the study Study participants who have consumed prescription medicine within 30 days before the study Study participants with neurological illnesses Study participants with chronic pain
	Study participants with psychiatric diagnoses
	Study participants with eczema, wounds, or sunburns on the sites of stimulation
	Study participants with a Body Mass Index of >30 kg/m^2^ or <18 kg/m^2^
	Study participants with contraindications to MRI^a^
	Study participants that decline information regarding potential pathological findings in relation to the MRI
	Study participants that have any kind of trauma resulting in pain and administration of analgesics in the period between experimental pain testing and MRI scan
	Study participants that experience a head trauma in the period between the experimental pain testing and the MRI scan
	

^a^Contraindications to MRI includes: claustrophobia, pacemaker implant, artificial heart valve, cochlear/stapes prosthetics, irremovable insulin pump, neurostimulator, metal clips from previous surgical procedures, other metallic foreign objects, shrapnel or shell splinter, catheters (eg, Swan Ganz), shunts and drainage tubes, and surgical procedures within 6 weeks before the study (subjected to individual evaluation).

Before the experimental day, each participant will be tested with the experimental pain models: BTS, heat pain detection threshold (HPDT), and pain during 1 minute of thermal stimulation of the skin (p-TS), as well as two psychological tests: Pain Catastrophizing Scale (PCS) and Hospital Anxiety and Depression Score (HADS; see Multimedia Appendix 1 and Clinicaltrials.gov identifier: NCT02527395). The results will be blinded to the investigator evaluating the MRI scans. Prior to, as well as on the experimental day, the investigator will interview all participants, and those that have one or more psychiatric or neurological diagnoses or illnesses will be excluded. All participants with prior history of psychiatric or neurological illness will also be excluded.

### Clinical Evaluations

#### Pain Testing

BTS, HPDT and p-TS are not performed as a part of this study per se, but all participants in the present study have been tested using these methods in a parallel non-imaging study (Clinicaltrials.gov Identifier: NCT02527395). Data concerning secondary hyperalgesia from this concurrent study will be extrapolated and included in the present study. The BTS method is briefly explained below. For further details, please refer to Multimedia Appendix 1.

#### Brief Thermal Sensitization

A computer-controlled thermode (Somedic MSA Thermotester; size 2.5 x 5 cm) is placed on the participant’s skin, centrally on the anterior part of the right thigh. The initial temperature of the thermode is 32°C, and the temperature is increased 1°C/second until it reaches 45°C. The temperature of the thermode remains a constant 45°C for 3 minutes, and while the thermode still has contact with the skin, the assessment of secondary hyperalgesia is conducted. The assessment of secondary hyperalgesia takes approximately 1-2 minutes, with a maximum duration of heat stimulation of 5 minutes.

#### Assessment of Secondary Hyperalgesia

The area of secondary hyperalgesia is quantified after stimulation with a 19G monofilament (von Frey hair) in 4 linear paths arranged in 90° around the center of stimulation. Stimulation will begin 15 cm from the center of stimulation and advance in steps of 5 mm with 1 second intervals towards the center of stimulation. When the participant states a clear change in sensation (ie, intense pricking, burning, tenderness) the place will be marked with a felt pen and the transverse and longitudinal axes will be measured for later area calculation.

#### MRI Scans

The MRI scans will be performed with a Siemens MAGNETOM Verio 3-tesla, with b17 software, and a 32-channel head coil. The specific MRI sequence and settings are detailed in Multimedia Appendix 2.

#### Anatomical MRI Scans

**Figure 1:**
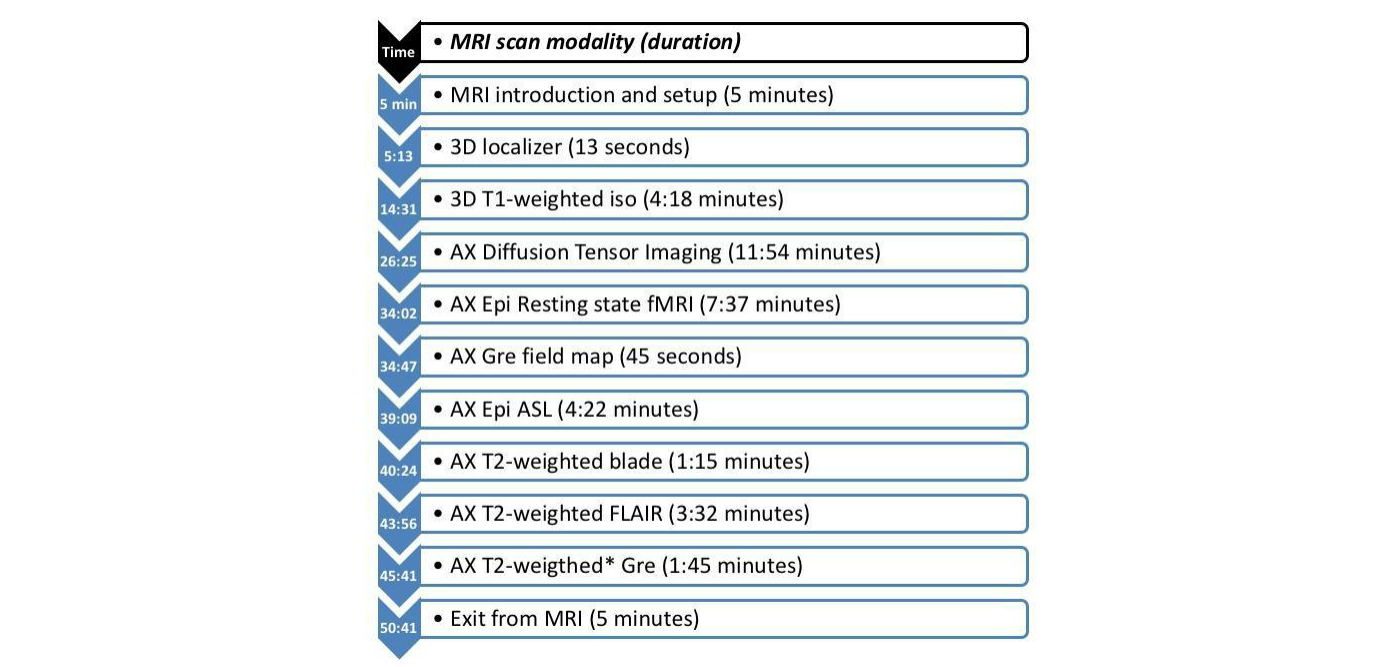
Schematic presentation of experimental day. Abbreviations: MRI, Magnetic Resonance Imaging; fMRI, Functional Magnetic Resonance Imaging.

T1-weighted images are recorded in order to construct detailed anatomical images of the brain. These images will be used to perform morphometry/volumetric analysis of cortical and subcortical structures in order to determine possible differences in the study population. To look for potentially confounding structural lesions and perfusion asymmetry, T2-weighted, T2-FLAIR, diffusion weighted imaging, gradient echo, and arterial spin labeling will also be performed as part of the imaging protocol (Figure 1).

#### Diffusion Tensor Imaging

Diffusion tensor imaging (DTI) is sensitive to the diffusion of water molecules. The net-diffusion of water molecules roughly occurs along the direction of the axons; hence, DTI can be utilized to perform white matter tractography (neuronal connections of the brain), or to detect changes in the white matter by using Tract-Based Spatial Statistics.

#### Resting State Imaging

In rs-fMRI the intrinsic connectivity networks of the brain are mapped by indirectly measuring so-called *spontaneous brain activity*. Rs-fMRI measures spontaneous changes in blood-oxygenation-level-dependent signal by detecting concomitant changes in magnetization that accompanies regional changes in brain blood flow.

#### Other Scans

As a supplement to the previously mentioned MRI scans, it is necessary to perform a number of technical MRI scans as well (ie, B0 field maps). These supplementary scans are necessary and important for later data analyses, and will not result in any further discomfort for the participants. For schematic presentation of the general procedure and the MRI scan sequence on the experimental day, please refer to Figure 1.

Participants will be informed that they must stay awake during the MRI scan period, and if they fall asleep they are obliged to inform the investigator. Participants that fall asleep during the MRI scan period will be excluded from the study.

### Outcomes

#### Primary Outcome

We aim to investigate the association between the volume of the left and right caudate nuclei and the size of the area of secondary hyperalgesia induced by brief thermal sensitization. The association will be expressed in adjusted and non-adjusted R^2^and prediction intervals for the area of secondary hyperalgesia, given fixed values of the caudate nucleus’ volume.

#### Secondary Outcomes

We aim to investigate the association between cortical and subcortical brain areas relevant for pain processing and the area of secondary hyperalgesia (primary somatosensory cortex, anterior- and mid-cingulate cortex, basal ganglia [putamen, accumbens nucleus, and globus pallidus], insula, and the cerebellum [12,42]). We will also use diffusion tensor imaging to investigate the association between the area of secondary hyperalgesia and white matter microstructure using tract based spatial statistics (TBSS) [43], and determine the connections between the pain-related areas, as specified previously using white-matter tractography.

#### Exploratory Outcomes

We will investigate the association between the volume of the left and right caudate nuclei and the following six parameters: (1) HPDT score, (2) p-TS max, (3) p-TS visual analog scale-area under the curve (VAS-AUC), and (4) the score of PCS-total, PCS-subscores, HADS-total, and HADS-subscores. When comparing healthy volunteers with a large (upper quartile) versus small (lower quartile) area of secondary hyperalgesia following BTS, we plan to investigate (5a) structural differences (volume) and the association with the size of the area of secondary hyperalgesia, (5b) differences in the association between area of secondary hyperalgesia and regions of interest extracted from TBSS, and (5c) differences in white matter microstructure in connections between pre specified pain related areas by white-matter tractography. In addition, we will examine (6) differences in resting state networks measured by rs-fMRI using independent component analysis (ICA; dual regression method).

### Hypothesis

The area of secondary hyperalgesia and the volume of the caudate nuclei are associated, with an inverse correlation (ie, a large area of secondary hyperalgesia is associated with a small volume of the caudate nuclei).

### Sample Size Estimation

Based on results from a previous study [11], sample size calculations are based on a Z-test of the Fisher transformed Pearson correlation. With a true correlation of r=-0.4 between the area of secondary hyperalgesia and the caudate nuclei, and a significance level of 2.5% to 5% according to the single step method (refer to *Statistical Significance*), a sample size of 52 is needed to obtain a power of 0.80 (β=0.20).

### Data Analysis Plan

#### T1-Weighted 3D Anatomical Images

Anatomical images will be pre-processed using the FreeSurfer imaging analysis suite version 5.3. FreeSurfer is a semi-automatic software package that performs volumetric segmentation of cortical and subcortical structures [44-46]. All volumes will be adjusted for total intracranial volume, and will result in volume estimates (mm^3^). This analysis is performed to avoid possible confounding results due to differential head-size between participants. The size of the area of secondary hyperalgesia will be blinded until completion of the T1-weighted 3D anatomical image analyses.

#### Diffusion Tensor Imaging

Diffusion-weighted images will be pre-processed using the Functional Magnetic Resonance Imaging Brain Software Library (FSL) software package version 5.0. This analysis includes corrections of potential head movement and inspection of image quality. The resulting data will then be processed via dtifit and tract-based spatial statistic. The re-aligned images will then be fed into bedpostx for local modelling of diffusion parameters. Using bedpostx in conjunction with TBSS allows for the modelling of crossing fibers. The re-aligned images will be fed into probtrackx2 for tractography between the anatomical areas mentioned above.

#### Resting-State Functional Magnetic Resonance Imaging

Rs-fMRI scans will be analyzed using FSL MELODIC. Pre-processing steps will include spatial smoothing, motion correction, high pass filtering, and brain extraction. In the pre-processing steps we will apply ICA using predefined (no less than 60) ICA’s without automatic dimension detection. Rs-fMRI scans will be co-registered initially to the individual T1-weighted 3D anatomical scans (after neutral flipping and brain extraction), and subsequently to the Montreal Neurological Institute (MNI)-152 brain atlas using non-linear registration. A full quality assurance will be performed consisting of inspection of head movement (<3 mm and <3 degrees), acceptable co-registration, and inspection of image quality.

Rs-fMRI data will be analysed using a model-free ICA and the dual regression method, as described by Abou-Elseoud et al [47], to determine differences in network connectivities with the whole brain. Standard, published brain networks in healthy participants will be used to identify the different networks using publicly available datasets from the Oxford FSL Group publication [48]. Once we have run an ICA analysis, the resulting components will be spatially cross-correlated with Oxford’s templates to identify those that better match the canonical networks described in the Oxford FSL Group publication [48].

Noise reduction will be performed manually. Randomized command with 10.000 random computations will be performed with a design matrix consisting of the unpaired t-tests (predefined high responders vs. low responders, and repeated with area of secondary hyperalgesia as co-variate). Results will be displayed upon the MNI-152 normal brain atlas after dimension change as *P*<0.05 after correction for multiple comparisons. Activated areas will be identified semi-automatically.

### Statistical Analyses

#### Statistical Analysis of Primary Outcome

The estimates of the volumes, from the T1-weighted 3D anatomical scans of the caudate nuclei (mm^3^), will be exported to a spreadsheet for regression analysis to assess the association between volume of caudate nuclei and secondary hyperalgesia areas. The association of the volume of the left and right caudate nuclei and the area of secondary hyperalgesia will be investigated by linear regression of the estimated best linear unbiased predictors (EBLUPS) on the individual volumes of the left and right caudate nuclei. The ability of the volume of caudate nuclei to predict individual variation in the area of secondary hyperalgesia will be investigated by linear regression on the EBLUPS as a function of the individual volumes of the caudate nuclei.

Significance of the caudate nuclei as a predictor will be assessed by analysis of variance (ANOVA) methods and the predictive ability will be quantified by summary of prediction error, including 95% prediction interval for the prediction of the size of the area of secondary hyperalgesia. Relevant examples will be presented illustrating how accurately the volume of the caudate nuclei predicts the area of secondary hyperalgesia in the individual participants or vice versa. The association will be expressed in R^2^and prediction intervals for the area of secondary hyperalgesia given fixed values of the caudate nucleus’ volume. All analyses of structural MRI data (both in the primary, secondary, and exploratory outcomes) will be adjusted for age.

#### Statistical Analysis of Secondary Outcome #1

From the T1-weighted 3D anatomical scans, volume estimates of the cortical and subcortical brain areas relevant for pain processing (mm^2^, mm^3^) will be exported to a spreadsheet for regression analysis to assess the association between volume of the relevant areas for pain processing and areas of secondary hyperalgesia. The association of the volume of areas relevant for pain processing and the area of secondary hyperalgesia will be investigated by linear regression of the EBLUPS in relation to the volumes of individual areas relevant for pain processing. The association will be expressed in R^2^and prediction intervals for the area of secondary hyperalgesia given fixed values of the volume of the relevant brain areas for pain processing.

Significance of the predictor will be assessed by ANOVA methods and in cases of significance the predictive ability will be quantified by summary of prediction error, including 95% prediction interval. Relevant examples will be presented illustrating how accurately the volume of the individual areas relevant for pain processing predicts the area of secondary hyperalgesia in individual participants, or vice versa. The association will be expressed in R^2^and prediction intervals for the area of secondary hyperalgesia given fixed values of the caudate nucleus’ volume.

#### Statistical Analysis of Secondary Outcome #2

The exact values of white matter structure expressed as the diffusion coefficient (Fractional Anisotropy value) of the relevant pain related areas will be extracted, and separate statistical analyses will be performed. To test the association between the white matter microstructure extracted following analysis using TBSS/DTI and the area of secondary hyperalgesia, individual measures computed from the TBSS and DTI will be investigated by linear regression.

#### Statistical Analysis of Exploratory Associations #1-4

From the T1-weighted 3D anatomical scans, volume estimates of the caudate nuclei (mm^3^) will be exported to a spreadsheet for regression analysis to assess the association between volume of caudate nucleus and the score of HPDT, p-TS-max, p-TS VAS-AUC, PCS, and HADS respectively. The association of the volume of the right and left caudate nucleus and HPDT, p-TS-max, p-TS VAS-AUC, PCS and HADS will be investigated by linear regression of the EBLUPS in relation to the volume of caudate nucleus.

Significance of the ability of the caudate nucleus’ volume as a predictor will be assessed by ANOVA methods. The ability of the caudate nucleus’ volume to predict individual variations in HPDT, p-TS-max, p-TS VAS-AUC, PCS, and HADS will be investigated by linear regression on the EBLUPS of the individual volumes of the caudate nucleus. Significance of the caudate nucleus as a predictor will be assessed by ANOVA methods and in case of significance, the predictive ability will be quantified by summary of prediction error, including 95% prediction interval. Relevant examples will be presented illustrating how accurately the volume of the caudate nucleus predicts HPDT, p-TS-max, p-TS VAS-AUC, PCS, and HADS in individual participants, or vice versa.

#### Statistical Analysis of Exploratory Associations #5-6:

Quantile-quantile plots and Shapiro Wilk’s test will be applied in assessment of normal distribution. If data are not normally distributed, Mann-Whitney U-test will be applied. If data are normally distributed, unpaired t-tests will be applied to investigate structural differences in brain volume, differences in white matter, and differences in rs-fMRI between the groups. To investigate potential differences in rs-fMRI, correction for multiple comparisons will be conducted by the MRI software using False-Discovery-Rate. Values of secondary hyperalgesia areas will be entered into the FSL-software, and a t-test will be performed.

### Missing Data

For all analyses, an intention-to-test analysis will be performed including all subjects that participated in the experimental day. Analyses will be based on all observed data. In case of missing data exceeding 5%, and indication of violation of *missing completely at random* by a statistically significant Littles test, a sensitivity analysis based on an appropriate model for *missing data at random* or *missing data not at random* will be performed.

### Statistical Significance

Earlier studies have demonstrated that the volumes of the right and left hemispheric caudate nuclei are associated with one another [11]. To preserve a family-wise error rate of 5% for the two co-primary outcomes of associations between the right and left caudate nuclei and the area of secondary hyperalgesia, we will adjust for multiple comparisons of the co-primary outcomes. The two *P*-values will be evaluated at a significance level between 2.5% and 5% according to the single step method. For this method the significance level is determined by the correlation between the two resulting test statistics. If they are perfectly correlated the result will be 0.05, whereas if they are independent it will be 0.025.

*P*-values in the secondary and exploratory outcomes are evaluated at a 5% significance level, and to adjust for multiple comparisons, *P*-values are adjusted by means of single-step correction [49]. In the analysis of rs-fMRI data, the software’s standard methods and False-Discovery-Rate will be applied for correction for multiple comparisons.

### Software

All statistical analyses not computed by the standard MRI software will be generated using the open source statistical programming environment R (R Foundation for Statistical Computing, Vienna, Austria).

## Discussion

We plan to investigate the association between experimental pain testing parameters and the volume, connectivity, and resting state activity of pain-relevant structures in the brain. We have designed this study to investigate basic pain physiology in healthy pain-free individuals, and we aim to investigate if any basic characteristics in the pain-free individual predict experimental pain responses. Current knowledge of basic pain physiology in humans is still sparse, and in order to compensate for the many factors that can affect pain responses, we have chosen to include a very homogenous population, consisting of healthy male volunteers between the ages of 18 and 35. The strict inclusion criteria diminish the influence of factors related to differences in the brain’s structure and function (eg, sex, age). Thus, this study aims to investigate aspects of basic pain physiology in healthy male individuals, and relevant caution should be taken when applying the results in a more heterogeneous population.

Our hypothesis, as well as our sample size estimation, are based upon previous results from Asghar et al [11]. Moreover, we wish to investigate if the volume of the caudate nuclei is associated with the size of the secondary hyperalgesia areas, indicating that this structure may be involved in the processes of central sensitization. Decreased volumes of the caudate nuclei have been demonstrated in a wide array of chronic pain patients, suggesting that these patients have a decreased ability to suppress and modulate pain stimuli [15,16,20-23,25]. With this study we plan to investigate if healthy participants with low volumes of caudate nuclei have a higher degree of central sensitization, and thus may be at a higher risk of developing chronic pain conditions.

The results of this study may also be informative for future studies and trials in the clinical setting, where pain remains a serious problem for postoperative patients. Knowledge of predictive factors and baseline pain dispositions in patients awaiting surgery may improve the design of trials on postoperative pain interventions, and improve our knowledge of the mechanisms responsible for the development of acute and chronic pain.

### Ethics

MRI uses a strong magnetic field and, unlike other imaging modalities, does not use radiation. MRI is non-invasive and has no known side effects. Due to the strong magnetic field, persons in the MRI room are not allowed to wear any metallic objects, and study participants will be informed of this prior to the MRI scans. Special MRI-compatible equipment will be used to monitor the participants inside the MRI scanner. Study participants will wear ear plugs and/or ear protection to avoid noise disturbances from the MRI scans, and will be equipped with an alarm call button in case of emergency.

A consultant radiologist will review all MRI scans. In the rare case of pathological findings, the radiology department at Bispebjerg and Frederiksberg Hospital has instructions for further treatment procedures. Briefly, the participant will be informed of the possible pathological findings and the participant will be scheduled for further radiological testing and referred to a specialist in neurology. Prior to inclusion in the study, participants will be informed of this procedure. If the participants do not wish to be informed of potential pathological findings, we reserve the right to exclude them before commencement of the MRI scan.

Likewise, the experimental pain testing conducted in the parallel study (Clinicaltrials.gov identifier: NCT02527395) do not cause damage to the skin or have any other long-term adverse effects. In rare cases, changes comparable with a first degree sunburn may appear. This study will be conducted in accordance with the principles of the Declaration of Helsinki. The protocol is approved by the local Danish Research Ethics Committees (identifier: H-15010473), and the Danish Data Protection Agency (identifier: RH-2015-149). This study is also reported on the international database (clinicaltrials.gov identifier: NCT02567318).

Informed written consent will be received from all volunteers before inclusion in the trial. Relevant provisions of the Research Ethics Committee regarding informed consent will be followed. Negative, positive, conclusive, and inconclusive test results will be published. We aim to publish the results in two separate publications, the first of which will report the primary and secondary outcomes, and the exploratory outcomes 1-4, and the second publication will report the exploratory outcomes 5 and 6.

## Multimedia Appendix 1

Other tests and evaluations.

## Multimedia Appendix 2

MRI-sequence.

## Abbreviations

3D: three-dimensional
ANOVA: analysis of variance
BTS: brief thermal sensitization
DTI: diffusion tensor imaging
EBLUPS: estimated best linear unbiased predictors
HADS: Hospital Anxiety and Depression Scale
HPDT: heat pain detection threshold
ICA: independent component analysis
MNI: Montreal Neurological Institute
MRI: magnetic resonance imaging
PCS: Pain Catastrophizing Scale
p-TS: pain during 1 minute of thermal stimulation
rs-fMRI: resting-state functional magnetic resonance imaging
TBSS: tract based spatial statistics
VAS-AUC: visual analog scale-area under the curve
