# Supplementary material for: Is the Volume of the Caudate Nuclei Associated With Area of Secondary Hyperalgesia? – Protocol for a 3-Tesla MRI Study of Healthy Volunteers
Source: JMIR Res Protoc. 2016 Jun 17;5(2):e117. doi: 10.2196/resprot.5680 (PMC4930528; doi:10.2196/resprot.5680)
Supplement: Supplementary file 1 [file resprot_v5i2e117_app1.pdf]

APPENDIX 2

Other tests and evaluations

The following tests and evaluations are not conducted in this study, but are conducted in the study: "*To determine the degree of association between Heat Pain Detection Threshold and area of secondary hyperalgesia following Brief Thermal Sensitization in healthy male volunteers"* (Clinical trials identifier: NCT02527395) that have previously been approved by the regional Committee on Health Research Ethics (protocol number: H-8-2014-012). Thus, the following descriptions are for informational purposes only.

Pain assessment

Pain is assessed with a Visual analogue scale (VAS), index from 0-100 mm, where 0 mm represents "no pain", and 100 mm represents "the worst pain imaginable".

Brief thermal sensitization (BTS)

A computer-controlled thermode (Somedic MSA Thermotester™; size 2.5x5 cm) is placed on the participant's skin, and the skin is heated to 45°C for 3 min. After 3 min., while the thermode still has contact with the skin, the assessment of secondary hyperalgesia is conducted. The test is conducted on the anterior right thigh.

Assessment of secondary hyperalgesia

The area of secondary hyperalgesia is quantified after stimulation with a 19G monofilament (von Frey hair) in 4 linear paths arranged in 90° around the centre of stimulation. Stimulation will begin 15 cm. from the centre of stimulation and advance in steps of 5 mm. with 1 second intervals towards the centre of stimulation. When the participant states a clear change in sensation the place will be marked with a felt pen and the transverse and longitudinal axes will be measured for later area calculation.

Pain during thermal stimulation (p-TS)

The computer-controlled thermode is placed on the participant's skin, and the skin is heated to 45°C for 1 min. During this the participant evaluates the pain with an electronic VAS-scale. The participant will continuously evaluate the pain using the electronic VAS-scale because of the fluctuations in pain intensity during this type of stimulation. The equipment automatically

calculates a VAS-score under the curve (VAS-AUC) and a maximum VAS-score for the time period. The participant will not be able to see the computer screen during the measurement, and each pain evaluation will be independent of the previous evaluation. The test is conducted centrally on the anterior part of the non-dominant lower arm

Heat pain detection threshold (HPDT)

Heat pain detection threshold represents the lowest temperature that is perceived as painful, when heating the skin with the computer-controlled thermode. The initial temperature is 32°C, and temperature is increased 1°C/sec. The participant is asked to press a button when the heat is perceived as painful. If 52°C (the maximum temperature for the thermode) is reached before the participant's threshold has been registered, the thermode will automatically return to the initial temperature of 32°C. The HPDT is calculated as an average of four stimulations. Each stimulation will be performed with an interval of 6-10 seconds. The test is conducted on the anterior of the dominant lower arm.

Hospital Anxiety and Depression scale (HADS)

HADS is a questionnaire consisting of 14 questions, and is a 4-point Likert scale with values from 0-3. HADS can be subdivided into HADS-A, evaluating anxiety, and HADS-D, evaluating depression. The highest achievable score is 42, and a total HADS-score will estimate the participant's level of distress. To evaluate anxiety and depression separately, HADS-A and HADS-D must be evaluated separately, with a maximum score of 21 in the two subtests.

The interpretation of the score in HADS-A and HADS-D is as follows:

- 0-7: Normal
- 8: Mild level of anxiety/depression
- 11-15: Moderate level of anxiety/depression
- ≥16: Severe level of anxiety/depression

The HADS questionnaire is to be completed before study day 1.

Pain Catastrophizing Scale (PCS)

PCS is a questionnaire consisting of 13 questions. PCS is a 5-point Likert scale with values from 0-4, and can be subdivided into 3 subtests, that each evaluates the central elements in catastrophizing:

Rumination, magnification and helplessness. The highest achievable score is 52, and with separate evaluation of the three subtests, the 3 different elements can be assessed individually. To evaluate the 3 elements separately the 13 questions must be evaluated in the 3 following subgroups:

- Rumination: The sum of question 8, 9, 10, 11. Maximal sum =16
- Magnification: The sum of question 6, 7, 13. Maximal sum = 12
- Helplessness: The sum of question 1, 2, 3, 4, 5, 12. Maximal sum = 24

The PCS questionnaire is to be completed before study day 1.
