# Supplementary material for: Is the Volume of the Caudate Nuclei Associated With Area of Secondary Hyperalgesia? – Protocol for a 3-Tesla MRI Study of Healthy Volunteers
Source: JMIR Res Protoc. 2016 Jun 17;5(2):e117. doi: 10.2196/resprot.5680 (PMC4930528; doi:10.2196/resprot.5680)
Supplement: Supplementary file 2 [file resprot_v5i2e117_app2.pdf]

APPENDIX 1

MRI Sequence

Anatomical MRI-scans

The following sequence will be applied: T1-weighted 3D FLASH (160 sagittal slices, matrix 256x256 mm, FOV 256 mm, echo time (TE) 2.98 ms, repetition time (TR) 2300 ms, Slice 1mm, in plane resolution 1x1mm, FA 9°); T2-FLAIR sequence (29 axial slices, matrix 256x256 mm, FOV 240 mm, TE 133 ms, TR 6570ms, Slice thickness (ST) 4 mm, TI 2133 ms, FA 130°.

The T2 TSE sequence (29 axial slices, matrix 256x256, 240 mm FOV, 4 mm ST with 0 mm gap,TR=6710 ms, TE =117ms, acceleration factor R=2,. The GRE hemo sequence (29 axial slices, 240-mm FOV, 4 mm ST with 0 mm gap,TR=450 ms, TE =19ms, acceleration factor R=2 matrix size 256 x 256, FA 20°.

Diffusion tensor imaging

The DTI sequence will be a Single shot EPI (240 mm FOV, 25 4 mm axial slices with 0 mm gap, TR=8000 ms, TE =93 ms, acceleration factor R=2 matrix size 256 x 256, b-value = 1000s/mm, diff directions = 64).

Resting state imaging

The resting state sequence will be a epi single shot (192 mm FOV, 42 axial 3 mm slices with 0 mm gap,TR=2250 ms, TE =26ms, acceleration factor R=2 matrix size 64x64, FA 82, measurements = 200.)

Pulse frequency and respiration frequency will be measured during the resting state imaging.

Arterial spin labelling imaging

The ASL will be Siemen’s 3D background suppressed ep2d pulsed sequence,(101 6mm axial slices, TR=2500 ms, TE=11ms, FOV 192 mm, voxel size 3x3x6 mm IR1=700 ms, IR2=1800 ms, acceleration factor R=2, FA 90°)
